# Intraoperative assessment of anastomotic microcirculation during right hemicolectomy with real‐time laser speckle contrast imaging is safe and feasible

**DOI:** 10.1111/codi.70162

**Published:** 2025-07-16

**Authors:** Rupan Paramasivam, Claudia Jaensch, Anders Husted Madsen, Mai‐Britt Worm Ørntoft

**Affiliations:** ^1^ Department of Surgery Gødstrup Hospital Herning Denmark; ^2^ Department of Clinical Medicine Aarhus University Aarhus Denmark; ^3^ NIDO | Centre for Research and Education Gødstrup Hospital Gødstrup Denmark

**Keywords:** anastomosis, colorectal surgery, decision support tool, laser speckle contrast imaging, microcirculation

## Abstract

**Aim:**

Successful anastomotic healing is essential in colorectal surgery and depends on adequate microcirculation at the resection site to prevent anastomotic leakage (AL). Traditionally, surgeons subjectively assess this. Laser speckle contrast imaging (LSCI) provides an objective, dye‐free, and non‐contact method for measuring the bowel end microcirculation that could perioperatively support the surgeon's assessment. This study aimed to determine the feasibility of LSCI and evaluate surgeons' subjective opinions on its potential to assist in surgical decision‐making.

**Method:**

This feasibility trial was conducted within the IDEAL framework as a non‐interventional multicentre study. Patients undergoing elective right hemicolectomy were included. LSCI measurements were conducted twice perioperatively: before and after anastomosis formation. Surgeons were blinded to all measurements. Postoperatively, LSCI images and a questionnaire were presented to the surgeon asking whether these images, if presented perioperatively, would have influenced the selection of the optimal anastomotic site.

**Results:**

High‐quality LSCI measurements were obtained in all 20 patients operated on by 17 different surgeons, with clear and interpretable perfusion images captured without compromising sterility or extending operating time significantly. The device was non‐invasive and added an average of 2 minutes to the total operation time. Fifteen of the 17 surgeons indicated that measures could have influenced surgical decision‐making. In 50% of the cases, the surgeon reported a change in the resection site, by an average of 1.2 cm, based on LSCI images.

**Conclusion:**

This study demonstrated that LSCI effectively displays colonic perfusion in real‐time without disrupting the surgical procedure. The potential clinical value of LSCI with additional visual feedback lies in assisting surgeons in selecting the most optimal anastomotic site, thereby potentially improving healing and surgical outcome.

**What does this paper add to the literature?**

Intraoperative assessment of anastomotic perfusion is subjective, impacting leakage risk. This study demonstrates laser speckle contrast imaging (LSCI) as a real‐time, dye‐free tool for objective intestinal microcirculation assessment. By influencing surgical decisions, LSCI shows potential to standardize perfusion evaluation in colorectal surgery, paving the way for future clinical research.

## INTRODUCTION

Colorectal cancer is one of the most common cancers globally [1], and surgical resection remains the primary treatment modality [2]. However, a significant challenge in colorectal surgery is the risk of anastomotic leakage (AL), which is one of the most severe and potentially life‐threatening complications following intestinal resection [3, 4].

Anastomotic leakage is widely considered a multifactorial complication. Factors such as the host immune response, local inflammation, and particularly the gut microbiome have recently been proposed to play critical roles in anastomotic failure [5, 6]. In addition, patient‐related variables including age, sex, smoking status, and body mass index (BMI), as well as technical and biological factors, all contribute to anastomotic healing [7]. Nevertheless, adequate blood supply to the anastomosis remains a primary prerequisite for successful healing; insufficient perfusion increases the risk of impaired healing and leakage [8, 9]. Research indicates that knowledge of the anastomotic perfusion may reduce the risk of AL, underscoring the need for reliable, objective tools to evaluate blood flow during surgery to improve patient outcomes [10].

Traditionally, the assessment of blood supply and tissue perfusion at the anastomosis relies on the surgeons' subjective evaluation of arterial pulsation, intestinal colour, and bleeding at the intestinal edge. However, these methods lack objectivity, quantitative precision, and predictive accuracy, and studies indicate that even experienced surgeons often underestimate the risk of anastomotic leakage [11, 12, 13].

Although several techniques have been tested for potential intraoperative use—such as transabdominal Doppler ultrasound [14], transabdominal laser Doppler flowmetry [15], and oxygen spectroscopy [11]—these have not been integrated into routine practice. Their limited integration is primarily attributed to technical challenges, high equipment costs, and a lack of reproducibility [16]. Emerging alternatives such as transrectal Doppler flowmetry have also shown potential, particularly in evaluating rectal anastomoses, by offering real‐time flow velocity measurements via endoluminal access [17]. However, its application is primarily limited to low rectal resections and requires additional intraoperative instrumentation, which may limit generalizability.

Thus, the most widely used technique for intraoperative perfusion assessment is currently Indocyanine Green (ICG) fluorescence angiography. ICG binds to plasma proteins and emits fluorescence under near‐infrared light, allowing surgeons to visually assess tissue perfusion. While ICG has proven valuable as a decision support tool and is well integrated in minimal invasive surgery, studies highlight several limitations [18, 19, 20]: These include the need for repeated dye administration; the timing of the visual assessment; and the indirect nature of perfusion assessment, as ICG tracks a contrast‐binding protein rather than direct blood flow itself [21]. Moreover, artefacts and variability in ICG signal interpretation can sometimes challenge its reliability [22], and the inherent time dependency has made standardization challenging [23]. Consequently, clinical studies using ICG have yet to demonstrate a consistent effect in reducing AL rates, rather than only a potential benefit [10].

Alternatively, LSCI offers promising perspectives. Unlike ICG, LSCI provides a dye‐free, non‐contact method to assess microcirculation directly by tracking red blood cell movement, enabling real‐time, continuous monitoring. This novel approach eliminates the dependency on contrast washout times and allows for repeated measurements without the need for additional contrast, thus supporting more flexible intraoperative decision‐making [24, 25]. Currently, LSCI is not widely available and has only been applied to abdominal surgery in small case series [26, 27].

Consequently, we wanted to conduct a feasibility study of LSCI to systematically evaluate its implementation in colorectal surgery, addressing factors such as ease of use, image quality, and the device's influence on surgical decision‐making. By using the IDEAL framework (Idea, Development, Exploration, Assessment, Long‐term Study), which is a structured approach to evaluate surgical innovations in a stepwise manner from initial concept to widespread clinical implementation, we designed a feasibility study, which is a crucial early‐phase assessment in the “Development” stage 2b, where new techniques are trialled to determine their practicality, safety, and potential impact on surgical practice [28, 29].

The aims of this feasibility study were twofold:

1. To assess the feasibility of LSCI in a clinical setting.
2. To evaluate surgeons' subjective opinions on whether LSCI could serve as a useful intraoperative tool to guide decision‐making, especially in determining the most favourable location for anastomosis formation based on real‐time perfusion data.

## MATERIALS AND METHODS

### Study design

This non‐interventional study in the Central Denmark Region was conducted at the surgical departments in three regional hospitals: Godstrup, Viborg, and Randers. A total of 20 patients undergoing elective right hemicolectomy for colon cancer were consecutively included. Patient records were reviewed for complications 30 days postoperatively.

### LSCI system

The LSCI device used in this study, Moor‐FLPI‐2 (Moor Instruments, Axminster, UK), is a Class 1 laser, classified as IIa under the Medical Device Regulation (MDR), meaning that it poses no risk during use and is CE‐marked as commercially available medical equipment. Numerous studies have confirmed its safety with no reported complications or side effects [30]. In this study, microcirculation was assessed using LSCI with a wavelength of 785 nm. The device was positioned perpendicular to the tissue surface at a fixed height of 25 cm, and each measurement was recorded over 30 s at a sampling rate of 25 fps. LSCI provides two types of measurement of the microcirculation: A colour‐coded image and quantitative measures for a given region of interest in the picture (“LSCI image”), and a quantitative measure reported in arbitrary units (laser speckle perfusion units, LSPU).

### Feasibility evaluation

Surgeons were blinded to the LSCI measurements, allowing procedures to be performed as standard of care. LSCI measurements were conducted twice intraoperatively: first, before anastomosis formation, with the surgeon marking the ideal resection line based on standard clinical practice, and second, after anastomosis completion. For each measurement, the following were recorded: the time required to perform the LSCI measurement, the number of measures needed to assure one high‐quality LSCI measure, and the number of breaches in sterility during measurements.

Feasibility was defined by a set of predefined criteria, including (1) technical usability of the LSCI device in the operative field, (2) ability to obtain high‐quality, interpretable images, (3) procedural safety including preservation of sterility, and (4) subjective surgeon feedback on its potential influence on intraoperative decision‐making. These parameters were selected to assess whether LSCI could be safely and effectively implemented during standard colorectal procedures.

### Subjective evaluation

Postoperatively, the LSCI images were presented to the surgeon, who then marked the optimal resection site *had* LSCI been available intraoperatively. The change in the location of the resection site was recorded in centimetres. To evaluate the surgeons' perception of LSCI, a custom‐designed questionnaire was completed postoperatively. The questionnaire consisted of six items targeting image interpretation, usability, and the tool's potential influence on intraoperative decision‐making. Surgeons were asked to rate how well they were able to identify transitions between well‐ and poorly perfused bowel on the LSCI images, as well as to evaluate the overall image quality. They were also asked to assess the ease of setup and whether they found the use of LSCI acceptable within the surgical workflow. Finally, the questionnaire explored whether the surgeons considered LSCI a potentially useful intraoperative tool and whether the images could have influenced their choice of resection site. Responses were recorded using a Likert scale (1–5) for the first five items, and a binary yes/no format for the final item concerning surgical decision‐making.

### Statistical analysis

All data was analysed using Stata software version 18 (StataCorp LLC, College Station, TX, USA). Patient demographics are presented as median with range (min–max).

To assess whether the proportion of surgeons who believed the tool could influence decision‐making was statistically significant, a binomial test was applied. Quantitative data were analysed using a paired *t*‐test.

A two‐sided *p*‐value of <0.05 was considered statistically significant.

### Ethics

This study adhered to the ethical principles outlined in the Declaration of Helsinki. Written informed consent was obtained from each patient prior to inclusion in the study. The study was approved by the Danish Medical Research Ethics Committee (Case No.: 2314383, Document No.: 2917373) and registered in the ClinicalTrials.gov public trials registry (NCT06637176).

## RESULTS

### Demographics and feasibility

The patient demographics are presented in Table 1. Twenty patients with right‐sided colon cancer were included between April 2024 and June 2024. Of the total cohort, 15 patients underwent laparoscopic right hemicolectomy, 4 patients underwent open right hemicolectomy, and 1 patient underwent extended laparoscopic right hemicolectomy. All anastomoses were made extracorporeally.

**TABLE 1:** Patient demographics.

Demographics	*N* (%)
Total patients	20
Male	8 (40%)
Female	12 (60%)
Age (years)	
Median	76
Range	46–83
BMI	
Median	28
Range	19–39
ASA‐score	
Median	2
Range	1–3
Procedure	
Laparoscopic right hemicolectomy	15 (75%)
Open right hemicolectomy	4 (20%)
Extended laparoscopic right hemicolectomy	1 (5%)
Operating time (hour:minutes)	
Mean	02:35
Range	01:41–03:51

*Note*: Presented as number (*N*) and percentage (%), and median with range.

**FIGURE 1:**
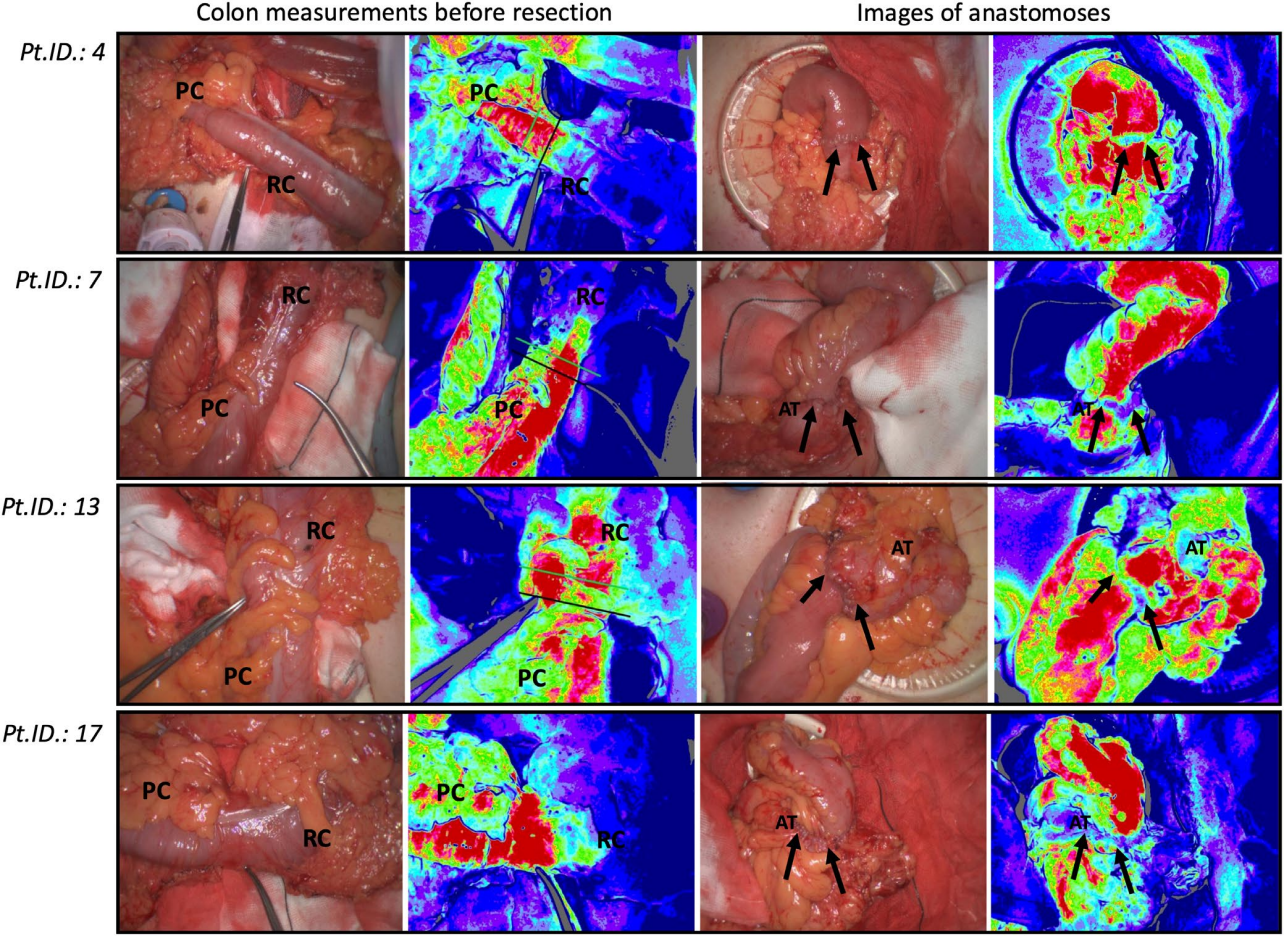
Examples of LSCI images for colon measurements and anastomoses with corresponding white light images. The LSCI images display a colour scale ranging from red to blue, indicating good to poor perfusion. For colon measurements, the black marked resection line (pean) represents the surgeons' intraoperative decision, while the green marked resection line indicates the desired adjustment based on the LSCI images. Images without any lines indicate that the surgeons maintained the resection site as initially marked by the pean. For anastomosis images, note the blue coloration of adipose tissue and sutures; however, it remains possible to assess blood supply directly adjacent to the anastomosis. RC, Resected Colon, planned for removal; PC, Preserved Colon, the remaining colon prepared for anastomosis formation; Black arrows: Anastomosis; AT, Adipose tissue; Pt.ID., Patient ID number. For a complete overview of all 20 cases from this study, please refer to Figure S1.

LSCI measurements were successfully obtained in all 20 patients, in the first attempt, except for one case, where two attempts were required. The use of LSCI prolonged the procedure by an average of 2 minutes per measurement, with no adverse events associated with the imaging device. No breach of sterility occurred. It was possible to obtain high‐quality images in all patients. However, in places with overlying adipose tissue, low perfusion rates were measured. Likewise, in areas of curved intestinal surfaces or peristalsis during bowel segment measurement, low perfusion rates were noted. Also, several artefacts were observed in the laser speckle pattern centrally in the anastomosis due to the sutures; however, it was still possible to assess the blood supply up to the anastomotic edge (Figure 1).

### Acceptability of LSCI

All 17 operating surgeons indicated that LSCI images were easy to interpret and they had no difficulty identifying the transition from well‐perfused to poorly perfused areas. All surgeons found LSCI acceptable to use during surgery.

When asked whether LSCI images could have influenced their choice of resection site, 15 of 17 operating surgeons responded “yes”; one surgeon responded “no”; and one surgeon responded “unsure”. This finding reached statistical significance (*p* = 0.001). Additionally, in 10 (50%) cases, the surgeon changed the optimal resection site by an average of 1.2 cm (range 0.27–2.37 cm) based on the LSCI images, while the remaining 10 (50%) did not alter the resection site. Among those who changed the resection site, six moved it in the anal direction and four moved it in the oral direction along the colon (Figure 1).

When surgeons were shown images of the anastomosis after formation, there was consensus that the LSCI image provided valuable quality assurance of the anastomosis microcirculation. However, none of the surgeons indicated that they would revise the anastomosis based on the images, even in cases of poor perfusion (Figure 1).

### Quantitative data

Quantitative data was extracted from the images and reported in LSPU on an arbitrary scale: The mean LSPU value at the primary resection line chosen by the surgeons was 1268 (95% CI: 1072–1464). At the secondary location, to which the resection was adjusted based on LSCI images, the mean LSPU value increased to 1416 (95% CI: 1040–1793), reflecting an improvement of 148 LSPU, though this difference was not statistically significant (*p* = 0.408).

### Postoperative surgical outcome

**FIGURE 2:**
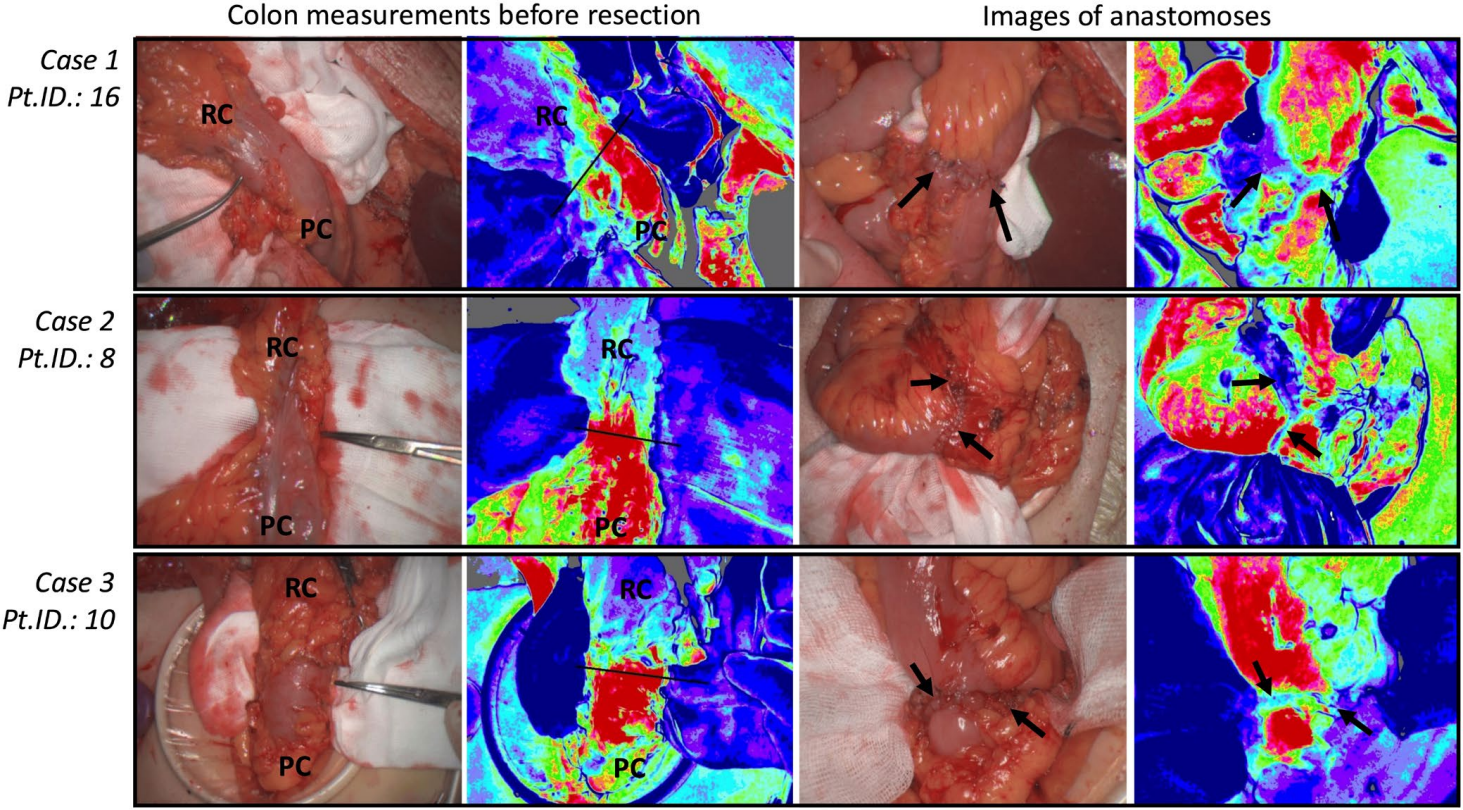
LSCI images from patients with complications. LSCI images of the colon before resection and the anastomosis, along with their corresponding white light images, are shown in the three cases with surgical complications. Case 1: Anastomotic leakage necessitating reoperation; Case 2: Reoperation due to bleeding, twisted small intestine in the anastomosis; Case 3: Small intra‐abdominal abscess close to the anastomosis (indicating AL grade A). For colon measurements, the black marked resection line (pean) represents the surgeons' intraoperative decision. In none of these cases did the surgeon find any reason to adjust the resection site. RC, Resected Colon, planned for removal; PC, Preserved Colon, the remaining colon prepared for anastomosis formation; Black arrows: Anastomosis; AT, Adipose tissue; Pt.ID., Patient ID number.

Three patients in the cohort developed complications. LSCI images from their intraoperative measurements are presented in Figure 2.

Case (1) One patient developed an AL, necessitating reoperation. Necrotic spots were found on the anastomosis, which prompted surgical breakdown of the anastomosis and formation of a stoma. Here, the intraoperative LSPU values at the surgeon's resection line were 653 LSPU, which is ~50% lower than the average LSPU values at the intraoperative resection sites in this cohort. However, the operating surgeon did not indicate any wish to change the resection site based on the feedback provided by the LSCI images.

Case (2) Due to bleeding, one patient required reoperation, during which it was discovered that the anastomosis was twisted on the small intestine site and required revision. The LSCI images of the anastomosis did not indicate reduced blood supply after the anastomosis formation.

Case (3) One patient was postoperatively found to have a small intra‐abdominal abscess close to the anastomosis (late abscess indicating AL, grade B [31]), which was successfully treated with antibiotics without further complications. LSPU values were 1615 at the resection line. Postoperatively, the surgeon indicated no wish to change the resection line.

## DISCUSSION

This study demonstrates the feasibility and potential clinical value of LSCI in colorectal surgery. LSCI proved both practical and safe for routine use, with no adverse events reported. In 50% of the cases, LSCI influenced the surgeon's decision to adjust the resection site, underscoring its potential clinical utility in guiding optimal anastomosis site selection. By involving multiple surgeons and centres, our study strengthens the evidence base and demonstrates that LSCI has a minimal impact on surgical workflow and receives positive feedback from surgeons regarding image quality and usability.

### Feasibility

The present study was conducted within the IDEAL framework's “Development” (stage 2b) phase and highlighted challenges that must be overcome before LSCI can be widely adopted into clinical practice. Specifically, the study was prospectively conducted with predefined feasibility outcomes (usability, image quality, safety, and impact on decision‐making) and included a consecutive patient series from multiple centres. Importantly, surgeons were blinded to the LSCI data intraoperatively, ensuring that patient care was unaffected by the use of LSCI. These elements align with the IDEAL recommendations for early‐phase surgical evaluations and provide a strong foundation for future exploration studies (Stage 3), where clinical outcomes and prognostic performance of the technique can be more rigorously assessed.

Previously, one Japanese study from 2020 also utilized the LSCI Moor system to assess perfusion during colorectal surgery [26]. However, key limitations to the study by Kojima et al. are the small number of patients included and particularly that a single surgeon performed all the operations. This introduces single centre, single surgeon bias and restricts generalizability, as outcomes may have been influenced by the surgeon's individual experience, skill, and interest in the outcome. In contrast, our study includes 17 surgeons across multiple centres, providing a more diverse and representative dataset. This multicentre approach mitigates surgeon‐related preferences and enhances the robustness of the feasibility evaluation. On the other hand, the multi‐surgeon design adds to the variability of the results and opposes the interpretation of results in regard to the prognostic potential of LSCI in a limited cohort like ours.

The present study demonstrated that LSCI was easy to integrate into the surgical workflow, with surgeons reporting no disruptions to the operative process and an average added procedural time of only 2 minutes per measurement. Unlike other methods that rely on repeated dye administration and time‐dependent imaging or require the presence of an additional specialist to perform measurements, LSCI can be operated independently by the surgical team. Additionally, LSCI involves a one‐time equipment investment (€33,500–$36,000) and provides real‐time, repeatable assessments without ongoing costs or delays, making it a cost‐effective and efficient alternative for intraoperative perfusion assessment.

### Acceptability

In the present study, the majority of surgeons (15 of 17) indicated that intraoperative availability of LSCI images could have influenced surgical decision‐making. In 50% of cases, surgeons reported that they would have adjusted the resection site based on the LSCI data, with an average shift of 1.2 cm. Although 1.2 cm may seem like a small adjustment, even minor changes can have a significant impact on patient outcomes. These findings suggest that LSCI provides valuable and actionable information that could meaningfully alter the surgical approach in a substantial proportion of cases, potentially improving patient outcomes. This is further supported by the quantitative data, which showed a trend towards higher perfusion values at sites where surgeons indicated they would have adjusted the resection based on LSCI data, although the difference was not statistically significant. Case 1 emphasizes this point; though the surgeon did not wish to adjust the resection site based on LSCI images, the analysis of the quantitative data revealed a very low LSPU value at the resection site. This suggests that LSCI might provide early warnings in such cases, potentially preventing complications. Moreover, LSCI offers the potential for objective quantitative measures, which could reduce variability in subjective evaluations. This, along with the capability for repeated measurements, distinguishes LSCI from other techniques, ICG included.

Future research should prioritize larger, prospective studies (“Exploration” stage of the IDEAL framework) to assess whether quantitative LSCI data can reliably predict complications related to poor microcirculation, such as AL. In time, this could lead to the establishment of a critical perfusion threshold, which again could pave the way for a more evidence‐based approach to surgical decision‐making. Looking forward, the integration of artificial intelligence (AI) in the interpretation of LSCI images may enhance the objectivity and clinical utility of the technology. Automated image analysis using AI‐based algorithms could reduce inter‐observer variability, assist in standardizing perfusion thresholds, and potentially provide real‐time clinical decision support. As LSCI generates both spatial and quantitative data, it lends itself well to machine learning models that could be trained to identify perfusion deficits associated with poor anastomotic healing. Future studies should explore this promising synergy between LSCI and AI‐driven image analysis to further advance intraoperative precision.

While our findings highlight the feasibility and clinical potential of LSCI for perfusion assessment, it is important to acknowledge that anastomotic leakage is not solely determined by perfusion; it is likely a multifactorial event [5, 7] influenced by factors such as the host immune response, microbial colonization, and tissue handling. Thus, while LSCI may aid in optimizing one critical component of anastomotic integrity—blood supply—it should be viewed as part of a broader strategy that includes attention to surgical technique, patient risk factors, and perioperative management. Future studies should consider integrating perfusion data with other biological and clinical variables to improve predictive models for anastomotic complications.

ICG is currently the most widely used method for intraoperative perfusion assessment in colorectal surgery and is well integrated into minimally invasive procedures. Several trials, including recent high‐quality studies [19, 20], have demonstrated that ICG may reduce anastomotic leakage in selected high‐risk groups, particularly in left‐sided or low anterior resections. Compared to ICG, LSCI offers several potential advantages. Most notably, LSCI is a dye‐free modality that provides real‐time, continuous assessment of microcirculation based on direct visualization of red blood cell movement. This eliminates the need for repeated dye administration and removes the time dependency inherent to contrast‐based techniques like ICG. In addition, LSCI enables repeatable measurements throughout the procedure and offers quantitative perfusion data, which may allow for more objective interpretation and future threshold‐based decision making. While ICG depends on subjective evaluation of fluorescence intensity and contrast timing, LSCI generates perfusion maps that can be numerically assessed and directly linked to tissue viability.

These characteristics position LSCI not only as a complementary alternative but as a potentially more standardized and scalable tool for intraoperative perfusion assessment than ICG. As LSCI technology evolves and becomes adaptable to laparoscopic workflows, future studies will be essential to directly compare its predictive performance against ICG, particularly in high‐risk colorectal procedures.

### Strengths and limitations

A key limitation of the current LSCI system (Moor) is its restriction to open or extracorporeal settings, which limits its applicability in fully minimally invasive procedures where intracorporeal anastomosis is increasingly favoured. This was the basis for the decision to evaluate LSCI during right hemicolectomy, along with its procedural consistency and the extracorporeal access to the anastomosis, which allowed for optimal imaging. However, recent studies such as the AVOID trial [19] and the EssentiAL trial [20] suggest that perfusion assessment techniques like ICG may have greater clinical impact in left‐sided and rectal resections, where leak rates are significantly higher. As the surgical approach in left side CRC treatment favours minimally invasive techniques, a prerequisite for the use of LSCI here is adaptation for laparoscopic use, recently demonstrated by early feasibility studies. Nevertheless, the necessary technology is still under development and not yet commercially available, so future technological refinements are needed to fully integrate LSCI into laparoscopic workflows and thus expand its clinical utility [27, 32, 33].

This study identified several technical and anatomical factors that affected LSCI image quality. Most notably, large volumes of overlying adipose tissue resulted in areas with artificially low perfusion values. This is likely due to scattering and absorption of the laser signal by fatty tissue, which attenuates the speckle contrast and reduces signal accuracy. Additionally, suboptimal angulation between the intestinal surface and the laser beam—particularly in curved, mobile, or peristaltic bowel segments—compromised the uniformity of image acquisition, occasionally resulting in shadowing artefacts and patchy perfusion maps. Based on our interpretation, these findings are consistent with the known optical behaviour of LSCI and emphasize the importance of controlled positioning and stable tissue presentation during acquisition.

Importantly, recognizing these challenges is consistent with the aims of the IDEAL framework at stage 2b, where feasibility studies are designed not only to test usability and safety but also to identify limitations in performance. Detecting where measurements are less reliable is essential to guide the refinement of the acquisition protocol and the future development of improved image interpretation algorithms.

Additionally, artefacts around sutures made it challenging to assess anastomotic perfusion accurately after formation, pointing to the need for further technological improvements of the method algorithm to reduce this interference. Also, the small sample size of this study limits the ability to draw definitive conclusions regarding the impact of LSCI on clinical outcomes.

The strength of the study is the use of the IDEAL framework, which ensures a strong foundation for future exploratory studies and the possibility for other researchers to transfer our results to their own surgical practice. The results from this feasibility trial increase the knowledge of LSCI and could lay the groundwork for subsequent phases that transfer LSCI closer to clinical implementation. Consequently, we plan to conduct a prospective study to advance LSCI to the “Exploration” phase, where the clinical efficacy, a critical perfusion threshold, and the value of LSCI measures in informing the risk of AL can be assessed, and compared to established methods like ICG fluorescence angiography.

## CONCLUSION

In conclusion, this study demonstrates the feasibility of LSCI in right hemicolectomies. LSCI was safe for patients and acceptable to surgeons and provided useful insight in the microcirculation. LSCI is a promising alternative tool for intraoperative perfusion assessment in colorectal surgery, though further research is necessary to fully establish the clinical benefits and optimize the implementation of LSCI in surgical practice.

## AUTHOR CONTRIBUTIONS

**Rupan Paramasivam:** Conceptualization; investigation; writing – original draft; methodology; data curation; formal analysis; project administration; writing – review and editing. **Claudia Jaensch:** Writing – review and editing; project administration; supervision; conceptualization; methodology. **Anders Husted Madsen:** Conceptualization; writing – review and editing; supervision; methodology. **Mai‐Britt Worm Ørntoft:** Conceptualization; writing – original draft; writing – review and editing; methodology; project administration; supervision; formal analysis.

## FUNDING INFORMATION

No specific funding was obtained for this study. Rupan Paramasivam is supported by grants from the NEYE Foundation, the Dagmar Marshalls Foundation, and NIDO Denmark. The funders did not play a role in the study's design.

## CONFLICT OF INTEREST STATEMENT

The authors have no conflicts of interest to declare.

## ETHICS STATEMENT

This study adhered to the ethical principles outlined in the Declaration of Helsinki. Written informed consent was obtained from each patient prior to inclusion in the study. The study was approved by the Danish Medical Research Ethics Committee (Case No.: 2314383, Document No.: 2917373) and registered in the ClinicalTrials.gov public trials registry (NCT06637176).

## Supporting information


Figure S1.

Appendix S1.

## Data Availability

All data generated or analysed during this study are included in this article and its Supporting Information files. Further inquiries can be directed to the corresponding author.
