# Supplementary figures and images for: Intraoperative assessment of anastomotic microcirculation during right hemicolectomy with real‐time laser speckle contrast imaging is safe and feasible
Source: Colorectal Dis. 2025 Jul 16;27(7):e70162. doi: 10.1111/codi.70162 (PMC12268114; doi:10.1111/codi.70162)

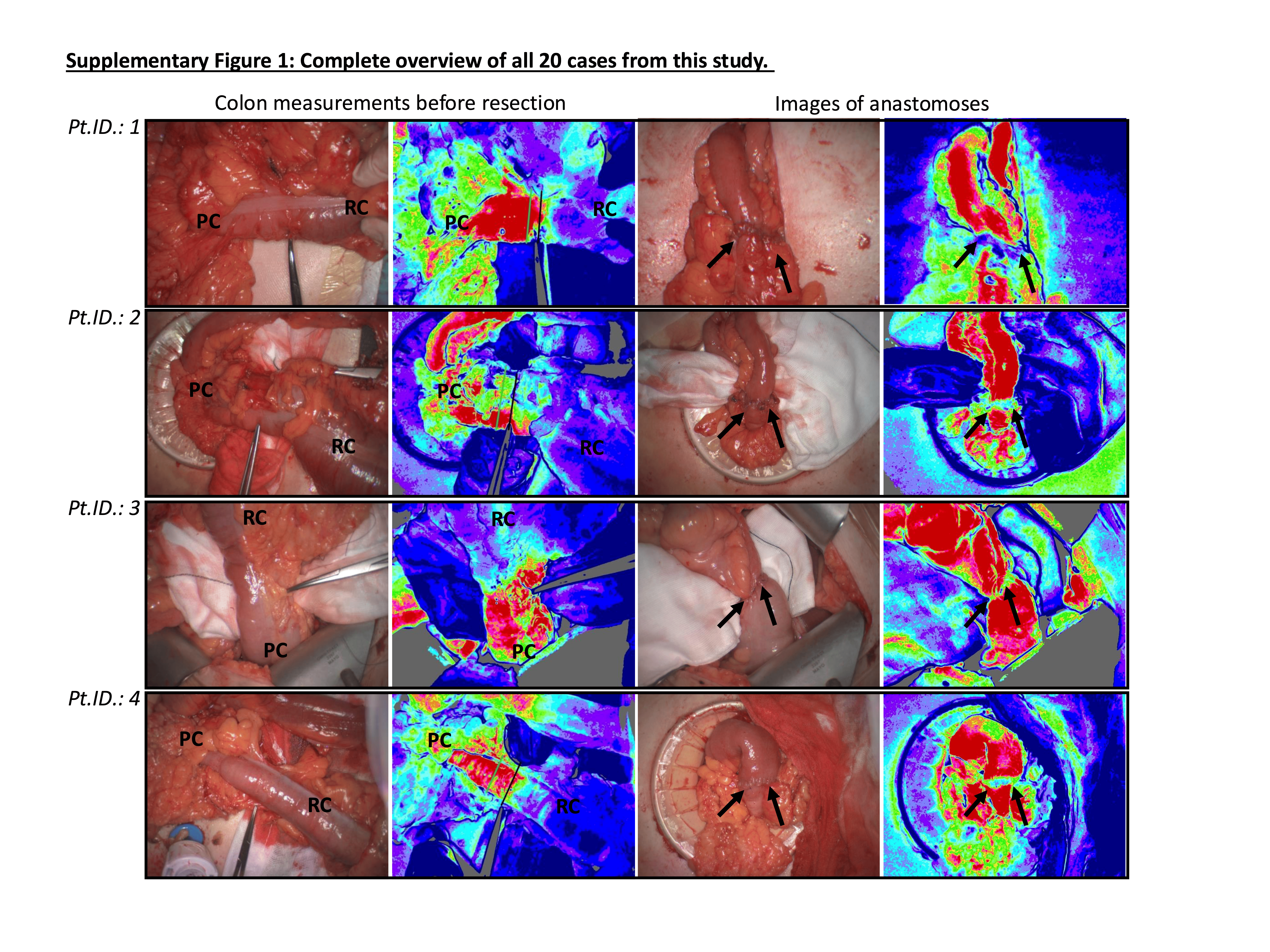

Supplement: Supplementary file 1 — Figure S1. [file CODI-27-0-s001.png]
