# Supplementary material for: Intraoperative assessment of anastomotic microcirculation during right hemicolectomy with real‐time laser speckle contrast imaging is safe and feasible
Source: Colorectal Dis. 2025 Jul 16;27(7):e70162. doi: 10.1111/codi.70162 (PMC12268114; doi:10.1111/codi.70162)
Supplement: Supplementary file 2 — Appendix S1. [file CODI-27-0-s002.docx]

**Supplementary Figure 1: Complete overview of all 20 cases from this study.**Each case includes an LSCI image of the colon before resection and the corresponding white light image. The black marked resection line (pean) represents the surgeons' intraoperative decision, while the green marked resection line indicates the desired adjustment based on the LSCI images. Images without any lines indicate that the surgeons did not find any indication to adjust the resection site based on the images and maintained the resection site as initially marked by the pean. Additionally, each patient's LSCI measurement of the anastomosis is shown alongside the corresponding white light image.
RC: Resected Colon, planned for removal; PC: Preserved Colon, the remaining colon prepared for anastomosis formation; Black arrows: anastomosis; AT: Adipose tissue; Pt.ID.: Patient ID number.
